# Comments on Environmental and Sanitary Aspects of the Scorpionism by *Tityus trivittatus* in Buenos Aires City, Argentina

**DOI:** 10.3390/toxins6041434

**Published:** 2014-04-22

**Authors:** Adolfo Rafael de Roodt

**Affiliations:** Laboratory of Toxinopathology, Center of Applied and Experimental Pathology, Faculty of Medicine, University of Buenos Aires/National Ministry of Health, Uriburu 950, 5 Piso, Lab. 555, Buenos Aires 1114, Argentina; E-Mail: aderoodt@gmail.com; Tel.: +54-11-4508-3602

**Keywords:** *Tityus trivittatus*, scorpion, antivenom, environment, distribution

## Abstract

Deaths by venomous animals are medical emergencies that can lead to death and thus constitute sanitary problems in some regions of the world. In the South of America, the accidents by these animals are a common sanitary problem especially in warm, tropical or subtropical regions, related with rural work in several countries. Argentina is located in the extreme South of South America and a minor part of the continental surface is in tropical or subtropical regions, where most of the accidents by venomous animals happen. However, in the big cities in the center and South of the country, with no relation to rural work, scorpionism, mostly due to the synanthropic and facultative parthenogenetic scorpion *Tityus trivittatus*, has become a sanitary problem in the last few decades. This scorpion is present in the biggest cities of Argentina and in the last decades has killed over 20 children in provinces of the center and north of the country, mostly in big cities. In addition, it seems that this species is growing and spreading in new regions of the cities. In this revision, some characteristics of this scorpion regarding its habitat, spreading in Buenos Aires city, combat measures and available treatments are discussed.

## 1. Introduction

Scorpions are among the animals most commonly implicated in human envenomation and death throughout the world. In Argentina, scorpions, spiders and snakes cause approximately 10,000 accidents per year, being scorpions responsible of around 60%–70% of the total cases [1,2], nevertheless, the mortality by scorpions is lower compared with that produced by snakes or hymenoptera [3].

In the country, over 60 different species of scorpions included in two Families (Bothriuridae and Buthidae) [4] have been described. Most of the Argentinean scorpions belong to the Bothriuridae Family, which is represented in the country by five Genera with more than 50 species. However, their sting does not have medical importance in humans. On the other hand, the Buthidae Family is represented by three Genera: *Zabius* (with two species), *Ananteris* (with one species) and *Tityus* with six species [4,5,6]. Scorpions responsible for envenomations and deaths of humans in Argentina belong to *Tityus* (*T*.) genus.

**Figure 1:**
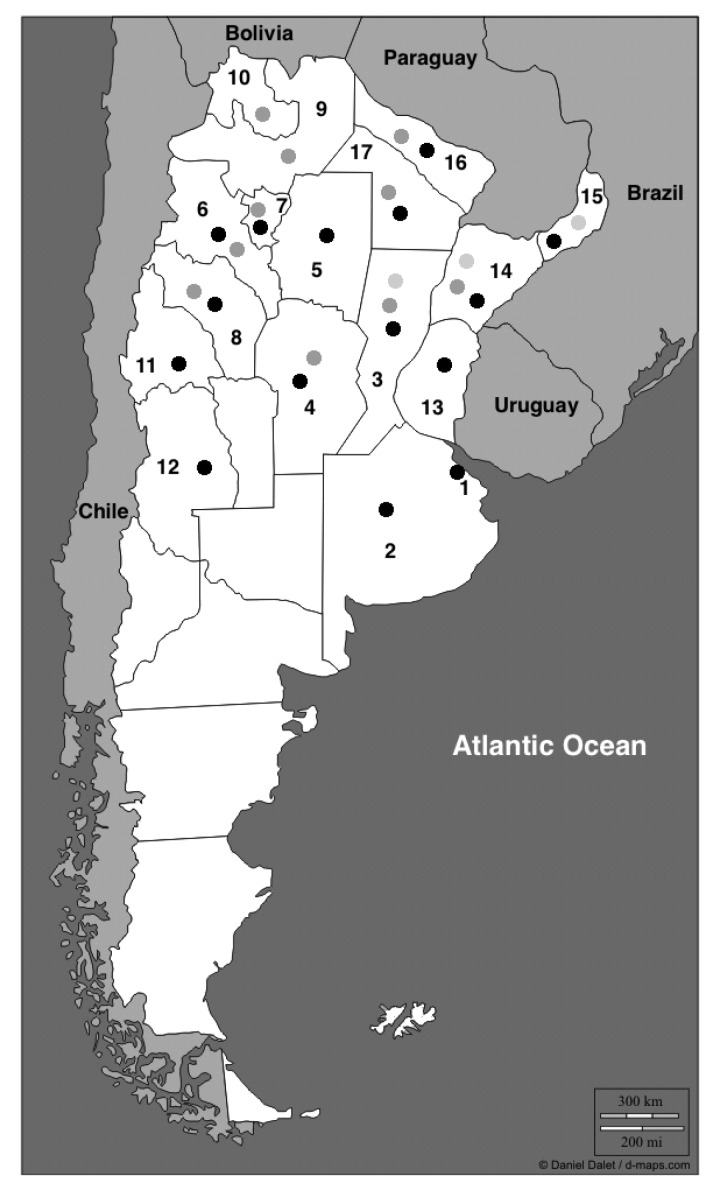
This figure shows the distribution of the three *Tityus* species of major medical importance in Argentina. *T. trivittatus* (black points), *T. confluens* (dark grey points) and *T. bahiensis* (light grey points). (1) Indicates the city of Buenos Aires and the other number the different provinces where these species of *Tityus* were found; (2) Buenos Aires; (3) Santa Fe; (4) Córdoba; (5) Santiago del Estero; (6) Catamarca; (7) Tucumán; (8) La Rioja; (9) Salta; (10) Jujuy; (11) San Juan; (12) Mendoza; (13) Entre Ríos; (14) Corrientes; (15) Misiones; (16) Formosa; (17) Chaco. Buenos Aires city (under the point indicating the presence of *T. trivittatus*, see Figure 3 and Figure 5), has a surface of 202 km^2^ and is limited to the South by a small river (Riachuelo) with the province of Buenos Aires, the province that surrounds the city to the West and North. The east of the city is the River Plate. The population is almost 3 million people with a density of around 14,000 inhabitants per km^2^.

**Figure 2:**
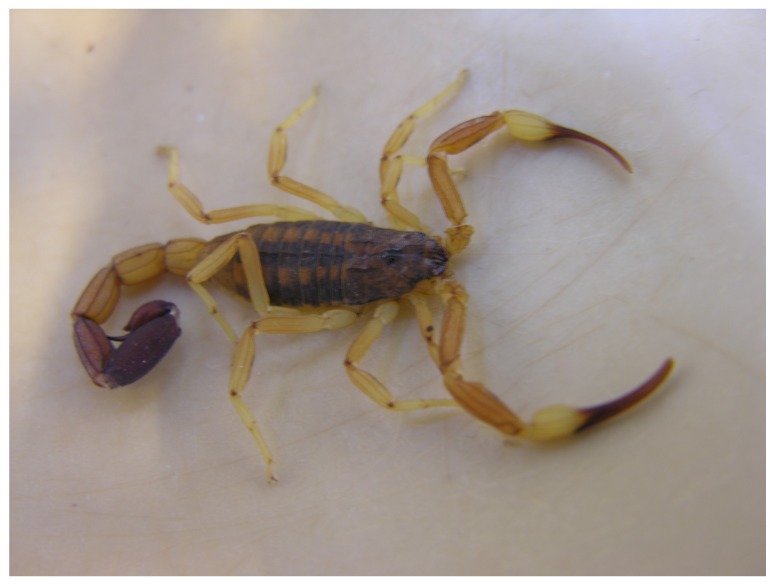
Adult specimen of *Tityus trivittatus*, showing its particular characteristics; thin and long pedipalps, three dark longitudinal lines in the back and the presence of sub-aculear apophysis.

Scorpions of *Tityus* Genus were described from the center to the north of the country and are represented by six species: *T. trivittatus*, *T. confluens*, *T. argentinus*, *T. uruguayensis*, *T. paraguayensis* and *T. bahiensis*, being *T. trivittatus* (Kraepelin 1898) the species with the widest distribution (Figure 1). Among these scorpions, *T. trivittatus* (Figure 2) is the most important due to the toxicity of its venom in most of the provinces [1,7] and number of accidents, with over 20 deaths since 1993 and most occurring after 2000 [7,8,9]. The sting by this scorpion is favored by the synanthropic behavior of this species [4,5,10,11]. The other scorpion responsible for deaths in Argentina is *T. confluens*, a species that killed four children between 2003 and 2010. [12]. This species became synanthropic in the last decades, being at present a problem of the same level of the *T. trivittatus* in some provinces of the North [12]. Some specimens of *T. serrulatus,* the principal species of scorpion with sanitary importance in Brazil, were reported in the north-east of Argentina, however up to date there have been only a few reports of its existence, and no accidents reported [13].

Although the mortality is low, the presence of *Tityus*, and specially *T. trivittatus*, in most provinces of the country represents a high risk due to the synanthropic characteristics of this scorpion.

In the present manuscript, we describe some historical data and new epidemiological data obtained from the population of *T. trivittatus* of the city of Buenos Aires, the most populated city of the country and where the venom of this species of scorpion has up to date showed low toxicity [14]. Nevertheless, the first envenomation that required treatment occurred two years ago [15], which indicates that attention must be focused on these envenomations despite the historical records of mild envenoming. In addition, aspects regarding the prevention, combat and treatment of envenomation are discussed.

## 2. *T. trivittatus* in the City of Buenos Aires

**Figure 3:**
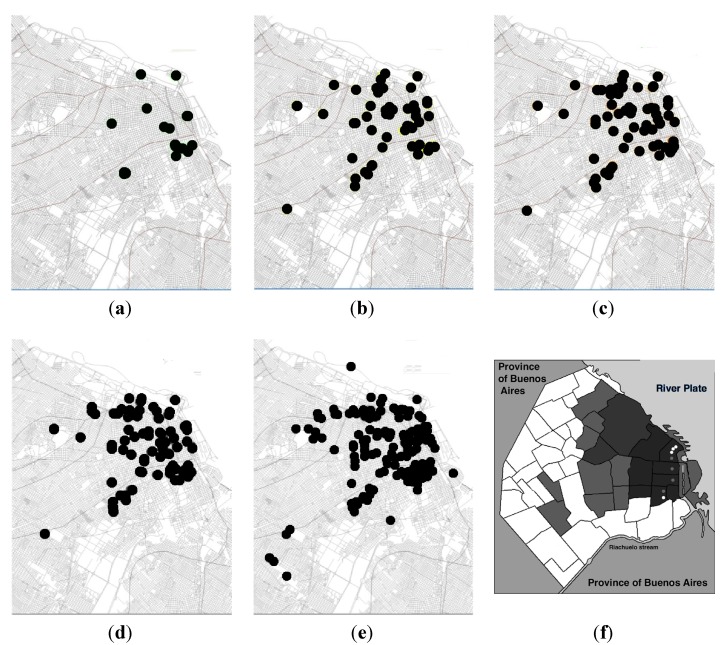
Spontaneous findings reported to one center during the period 2001–2011. Black points indicate the point where the scorpions were found. (**a**) Period 2001–2002; (**b**) 2001–2004; (**c**) 2001–2006; (**d**) 2001–2008; (**e**) 2001–2011; (**f**) indicates in dark the neighborhoods with the highest number of scorpions found during the period; in dark grey the neighborhoods with medium notifications; and in grey the districts with a minor amount of notifications. Light grey indicates no notification in this period. White points in the right of the figure indicate the biggest train stations of the city (terminal) and grey points, metro terminal stations in these districts. Lines in Figure 3a–e represent the main course of metro and train railways (not all) of the zones where *Tityus trivittatus* were found. Data from [16] and [17].

*Tityus trivittatus* is a scorpion that can be found from the center and south of Brazil to the center of Argentina. Possibly, the scorpion colonized the center of Argentina, and especially, the big cities like Buenos Aires when it was transported with the wood used for the construction of train and metro stations [11]. The first studies on *T. trivittatus* distribution in Buenos Aires suggested a relation between the finding of this scorpion and the metro railways [10,11], which was confirmed 20 years later with new studies [16,17] (Figure 3). In a big city like Buenos Aires, with scarce natural predators and the “protection” of the basements, underground galleries, underground water or electric chambers, underground metro railways, building plumbing (specially in old buildings) and other architectonic characteristics of the constructions, especially in the oldest regions of the city, this scorpion can very easily survive. The same situation is observed in other big cities like Córdoba, Rosario, Santa Fe, Santiago del Estero, San Fernando del Valle de Catamarca, La Rioja, Tucumán and others. All the same, in small populations it is also possible to find this scorpion, although to a lesser degree of findings and numbers of different specimens. The survival of these populations in the big cities is favored by its reproductive characteristics. It was proven that *T. trivittatus* is a facultative parthenogenetic scorpion [18,19]. This reproductive strategy undoubtedly greatly increases the opportunities for dispersion and invasion of anthropogenically-impacted habitats, like cities with big constructions and networks of metros and trains, and it was observed with other species of *Tityus* which show an important plasticity in adapting to new habitats [19,20,21].

## 3. Increasing Findings of *Tityus trivittatus* in Buenos Aires City

### 3.1. A Comment on Finding T. trivittatus and Accidents Resulting from this Species of Scorpion

As mentioned, *T. trivittatus* is present in most big cities of the country, where its synanthropic characteristics, adapting to live in human constructions and environments, favors the contact between man and scorpion, as described with other species of *Tityus* [20,21]. With the exception of Buenos Aires, fatalities have been recorded in other big cities of Argentina.

In recent years in Argentina, the number of notifications of scorpion findings increased as well as the number of accidents. During the period 1993–1999, only three fatalities throughout all the country were recorded [7] whereas in the period 2000–2006, 19 deaths were recorded [9]. From 2004 up to date, several deaths and numerous severe envenomations have occurred. This is disturbing since in the country, up until the 1980s, accidents by scorpions were known, but fatalities were unknown. This change in the number of accidents and notifications is worrying. Nevertheless, notifications of scorpion findings can be much influenced by the sensation of the population after a death or a “cataclysmic” advertising in the press [22]. On the other hand, the number of envenomations by *T. trivittatus* sting that causes severe cases and deaths in provinces, where no records of severe envenomation exists, turned this situation into a problem that deserves to be studied. In this regard, in the city of Buenos Aires, where any moderate or severe case was historically registered, in December of 2009 a case of a girl that required treatment was registered [15]. In addition, in other provinces, a fatality of a teenager was registered and an adult with a severe envenomation was presented, constituting the first severe accidents and death in “non-children” patients.

The venom toxicity of this scorpion in the city of Buenos Aires, up to date, is lower regarding the toxicity of the same scorpion in other regions of the country. The necessary dose to kill a mouse is around two to 10-fold higher than the venom of *T. trivittatus* from other regions of the country [14], which is in accordance with the (generally) mild course of the envenoming in this city [8,15]. However, the increase of the area where these scorpions can be found [16,17] indicates the need to focus the attention to prevent potential accidents in zones of the city where the presence of *T. trivittatus* was not historically registered.

### 3.2. Finding of T. trivittatus in the City of Buenos Aires

In some studies on the distribution of findings of *Tityus trivittatus* [16,17], we observed that the population of this scorpion has expanded from its geographical area of influence during the last decade in Buenos Aires city [11,22] and there also exists an increase in the frequency of cases [7,9].

**Figure 4:**
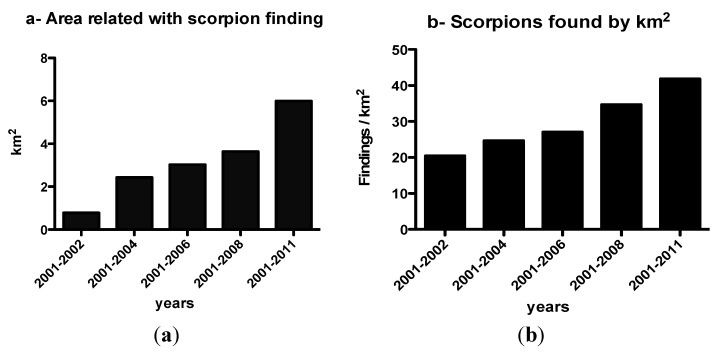
This figure shows the increase in the areas where this scorpion is found. (**a**) In km^2^ the surface affected by the findings in relation to the years; (**b**) The number of scorpions by km^2^ found in relation to the years. Data from [17].

Due to the occurrence of envenomation with severe cases and deaths in the country [8], we registered the findings of this scorpion in Buenos Aires city in order to know its present distribution. The records from spontaneous consultations for scorpion findings kept by the Research and Development Area of the National Institute for Production of Biologicals “Dr. Carlos G. Malbrán” of the Ministry of Health were studied several times [16,17,22] in order to compare the findings with previous observations [11]. Findings were classified by date and location, further geo-referenced in a digital map and analyzed in a geographic information system (GIS). These findings were differentially plotted for increasing time intervals from 2001 to 2011 [16,17]. The total area associated with reported findings in km^2^ and the number of events per km^2^ was computed for each time interval. The geographical area within Buenos Aires city where *T. trivittatus* findings have been reported, increased about 0.5 km^2^ per year since 2001 and the findings showed an increase to the west of the city [17] (Figure 3 and Figure 4). Despite the increasing number of findings of *T. trivittatus*, the number of cases of scorpion stings in Buenos Aires city did not increase and no deaths were recorded [2,3,23].

**Figure 5:**
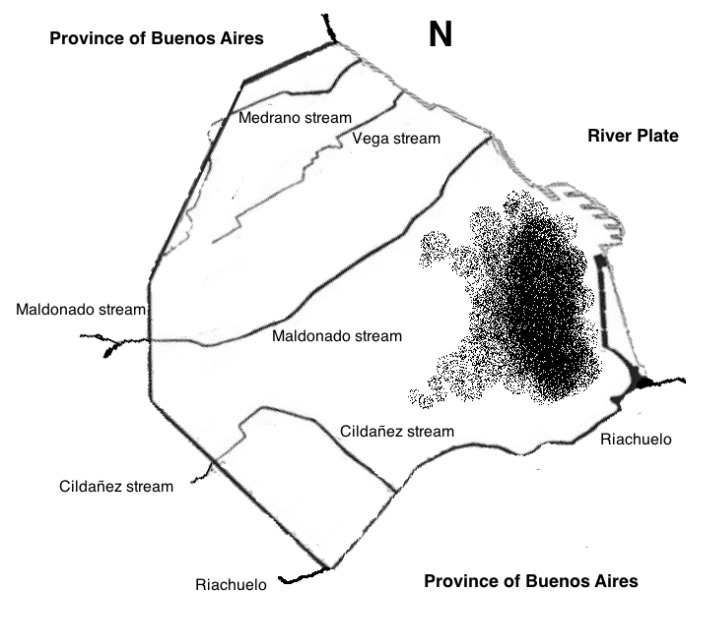
Rivers (River Plate and Riachuelo) and the biggest streams of Buenos Aires city: Maldonado, Cildañez, Vega and Medrano (all these tunneled). The dotted zone indicates the region of the city where the highest numbers of scorpions are found up to date. The absence of a relation with the main courses of water is clear.

The pattern of the findings and distribution is mostly related with trains and metro railways (principally at the east of the city) and with the older constructions of the city. No relation with the subterraneous courses of water was found, which are mainly located in the south and north of the city (Figure 5).

**Figure 6:**
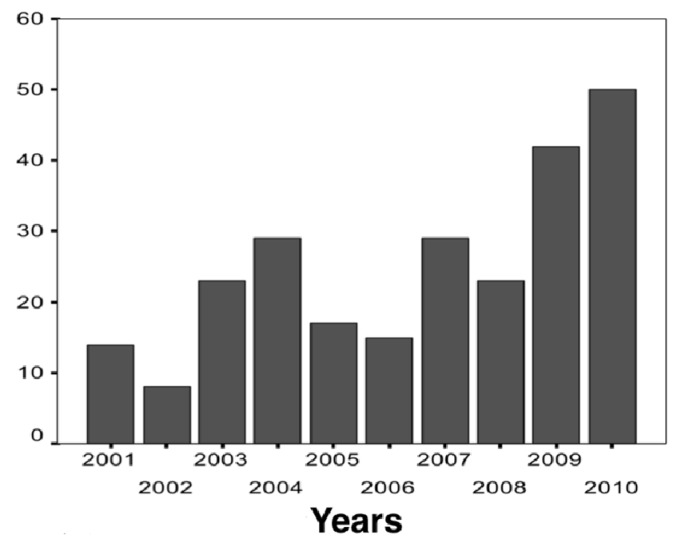
This figure shows the number of *T trivittatus* found and spontaneously reported by year from 2001 to 2010.

Over the entire period of study, the months with the highest number of findings were October to April, the warmer months. In the months of July, August and September, the lowest number of findings, were registered while in the months of November to December and January to February (end of Spring and Summer) the highest number of findings were produced [23]. The frequency of findings was significantly greater in the last two years (Figure 6).

### 3.3. Type of Neighborhoods Mostly Populated by T. trivittatus up to Date

The areas with a higher density of findings are those in relation to the oldest and most populated neighborhoods of the city: Retiro, Recoleta, San Nicolas, Monserrat, Constitution, San Telmo, San Cristobal, Balvanera, followed by Palermo and Villa Crespo, and surrounding neighborhoods in the east, north-east and south-east of the city. In these zones, very recent edifications coexist with the oldest edifications, and most important: four big terminals of train stations (three in the North, one in the South) and the terminals of five metro lines and its interconnections (between metro lines and train lines) are located in the East (See Figure 3f). Furthermore, during the period studied there were also scattered findings in other regions of the city (Figure 3). There was an increase in spontaneous reports of findings and in the entire period, the tendency was positive. In the last two years, the number of reports was over two-fold when compared with the former frequencies (2001–2008) (Figure 6) and relationships between the finding of *T. trivittatus* with lines of trains and underground metro railways, especially the oldest ones, were observed. These findings are congruent with the theory of the introduction of the scorpions to Buenos Aires city, which would have arrived in wood from the north of the country through the railways and train stations in the beginning of the 20th century [11]. These are located principally at the east of the city in concordance of the zones with higher density of findings [16,17,22] (Figure 3 and Figure 4). However in the last years scorpions begun to be found in the west and southwest, as can be observed in Figure 3a–e.

Regarding the places where the specimens were found, scorpions were found either in private houses or buildings or in institutional buildings. In buildings, specimens were found underground, or on the ground floor and upper floor, the maximum level which the scorpions were found being the 13th floor (two reported incidents). When the findings were made in institutional buildings or in private constructions, a relation with warm and humid places was observed.

## 4. Comments on Experience in Active Finding of *T. trivittatus* in Different Cities of Argentina

Although not common, in some occasions, the active capture of *T. trivittatus* was done, generally as a result of requests from sanitary authorities. These captures were quite contrasting in the different provinces due to the different characteristics of weather and geography. For example, in the provinces of the North, scorpions can be found in houses and constructions. In addition, they can be found around the house and in gardens associated with objects like pots, flowerpots, furniture, stones in the ground, piles of trash, *etc.* In big cities like Buenos Aires, Córdoba, Rosario *etc.*, scorpions are mostly found into human constructions [23]. In our experience of active finding, scorpions in Buenos Aires are mostly found underground in old constructions, preferably in warm and humid places and associated with cracks in the walls, steam tunnels, water pipe tunnels, chambers for electricity, boilers, *etc.* In our experience, the repeated “hunting” for scorpions brings similar results regarding the number of scorpions observed and captured. We could find in a single edification with several buildings in its property, scorpions with a density index (scorpions found in environments/environment examined) around values of 2, 5 in the first capture and of around 3, 0 two weeks later. In agreement, other groups, which in different active searching for *T. trivittatus* in buildings of Buenos Aires city observed that the number of scorpions were very similar in the different captures. This persistence of the scorpions could be related to the complicated and anfractuous places where these animals inhabit the buildings, being specially the old constructions with plenty of different characteristics that favor the survival of these animals. For these reasons the maintaining and spreading of this scorpions in cities with buildings and underground routes of these characteristics is explainable considering *Tityus* scorpion’s high plasticity in adapting to new habitats of [18,19,20,21].

## 5. Control and Prevention

Due the characteristics of *T. trivittatus* in Buenos Aires and other big cities, the control of the populations of scorpions becomes complicated. In fact, the common measures to control arthropods or arachnids do not totally represent the best choice to avoid accidents by this scorpion.

The control of *Tityus trivittatus* must focus on preventing the contact between humans and scorpions and the reduction of the number of scorpions. These can be done through: active search and capture or killing of the scorpions (difficult or impossible in some buildings), the use of chemicals (difficult and without positive results at present), introduction of natural predators (almost impossible in big cities), environmental-architectonic measures or, depending the situation, through combinations of the former.

### 5.1. Chemical Combat

Although the use of chemical products is one of the main measures adopted for controlling scorpions [24], few studies have focused on the efficacy and viability of this approach. The main measures taken for the control of scorpions naturally occur in the localities where they are regarded as a serious hazard to public health and consist on the intra and extra domiciliary fumigation with chemicals, which can be done only in some type of human environments. Nevertheless, studies are scarce, and in the case of *T. trivittatus*, inexistent. Due the biological characteristics of this scorpion, the combat with chemicals is very difficult and no positive results have been proven in Buenos Aires and in other cities where *Tityus* scorpions inhabit. Regarding other species of *Tityus*, the use of chemicals was not positive, being dangerous to humans and other animals or did not bring a solution to the problem. For example, in the combat of *T. stigmurus*, the fumigation showed a dispersive effect on the scorpion population [25,26], with no significant improvement in the reduction of the number of scorpions [27]. In addition, an excitatory effect on scorpions was observed after treatment with some chemicals in Argentina [27,28]. Although in Mexico a reduction in the number of *Centruroides* and *Vaejovis* was observed with high doses of pyrethroids, these scorpions could not be totally eliminated, due the difficulty of treating all their refuges [29], although some utility could be observed in the short term when the number of scorpions is elevated [30]. The same problem regarding the difficulty to reach the nucleus of scorpions using chemical products will be the case for the use of these products against *T. trivittatus* scorpions. Nevertheless, when the fumigation can be used with positive results, some pyrethroids seem to show activity on some species of scorpions like *Centruroides* [29].

However, the Scorpion Control Manual, from the Brazilian Ministry of Health [31], makes mention of the reasons by which the chemical treatment against *Tityus* scorpions is ineffective for its control. The habit of these animals to shelter in the cracks of walls, underneath cardboard boxes, and in piles of bricks, tiles, pieces of wood, and fissures or crevices in the ground, together with their ability to remain for months without moving, are several of the reasons that “protect” the populations from fumigation. Additionally, *Tityus* scorpions have the supposed ability to remain with their pulmonary stigmata closed for a long period. Regarding the products, the same Manual does not recommend the use of chemical domestic sanitation items consisting of formaldehyde, cresols and para-chlorobenzenes and products used as insecticides, raticides, cockroach-killers or repellents of the pyrethroids and organophosphates groups. This is because, according to the Manual, the displacement and consequent dispersion of the scorpions from the places previously not exposed to the effects of these products increases the risk of attacks. Also, it could create a false feeling of being protected on the part of the residents, who will be led to believe that the problem has been overcome and prevent them from coming to terms with their environment. The Scorpion Control Manual states that, up till now, the effectiveness of the chemical products for scorpion control in the natural environment has not been scientifically proven. When there is a need to control cockroaches in places where scorpions are present, the use of formulas with gel or powder is recommended. Until some study is done specifically on *T. trivittatus*, these suggestions seem to be the most rational and effective for controlling this scorpion.

### 5.2. Control with Predators

The use of natural predators has been suggested for the combat of *Tityus* scorpions. There are several species, natural enemies of the scorpions, like frogs, monkeys, bats, owls, some rodents and spiders and other domestic animals that can be of use for combat in gardens or around houses, when it is possible, like chickens or ducks [23,30,31,32,33,34]. However, this method is not widely published in scientific literature [30]. Although chickens are very active during the day and scorpions at night, chickens when looking for food like arthropods or worms can eat scorpions located in cracks under stones, leaves or debris. Although it is possible to do it in certain cities or towns, it is impossible in other type of cities, especially with buildings with architectonic restrictions, which is the case of Buenos Aires city. In Brazil, due to a serious scorpionism problem, the authorities of an affected zone distributed chickens in the population, but its efficacy could not be evaluated [30]. However, this type of combat in Buenos Aires city would not be useful due to the environment in which these scorpions can be found. Nevertheless, a point must be mentioned, in constructions where *T. trivittatus* lives together with cats, scorpions are only found in environments that are inaccessible to the cats, an observation of the author with Dr. Andrés Ojanguren-Affilastro.

### 5.3. Localization and Capture

Although this procedure when combined with others could bring some results, in the edifications of big cities and buildings this becomes impractical or impossible and the active search for scorpions is not recommended as a method to be used alone for controlling scorpion populations. In our experience, this method in cities like Buenos Aires is ineffective, especially in big and old constructions.

### 5.4. Measures for Prevention in Houses and Buildings

All these measures are directed at avoiding the contact human-scorpion, and are especially useful in buildings where other types of control would be impossible. The repair of cracks in floors and walls, broken pipes, garbage disposal, daily cleaning and use of safety covers on drains, are measures to be taken prior to any other method of combat. To close or cover with sanitary tape blocks the drains or pipes from which scorpions could enter in the rooms inhabited by humans. Only after taking these measures, other combat measures to reduce the burden of animals should be considered.

### 5.5. Combined Methods

The analysis of the situation, frequencies of scorpion findings in different regions and locations determining its special distribution is essential for their control. The participation of the population and training of sanitary staff on scorpion characteristics and control measures are fundamental to undertake effective methods of control and prevention. The defined search and removing of the scorpions from dwellings, and environmental measures in constructions, makes the population of the zones of risk be aware of scorpion envenomation and the ways to avoid or reduce the possibility of accidents. Care may be taken in architectonic measures and basic measures to avoid the entry of scorpions into environments inhabited by humans in the different types of constructions. This is particularly important in buildings where it is impossible to reach the nucleus of scorpions to eliminate them.

## 6. Treatment

Since the first report, presented by Sancón in 1928 [11], it is known that the main clinical features of scorpionism by *T. trivittatus* are pain that occur in almost all affected people while severe systemic manifestations (e.g., sweating, hypertension, vomiting) as well as more severe organ/tissue dysfunction (e.g., cardiorespiratory manifestations) are fortunately uncommon. Thus, scorpionism by *T. trivittatus* (and recently by *T. confluens*) being a problem of public health with no definitive solution constitutes a risk that deserves better attention in order to diminish the mortality and morbidity as much as possible.

Since the severe envenomation and deaths by *Tityus trivittatus* are relatively new, there is not an abundance of bibliography regarding the clinics of the envenomation and treatment by *T. trivittatus* and *T. confluens*, with only few descriptions of clinical cases [15,35,36,37,38,39,40,41].

The appropriate treatment of scorpion envenoming was controversial. Several authors consider that a correct clinical management of Buthidae envenomation makes the use of specific antivenom unnecessary [42,43,44,45], while other authors do recommend the use of the scorpion antivenom [46,47,48,49,50,51,52,53,54]. However, in a recent clinical study it was proved that the use of antivenom is more effective than the pharmacological treatment alone to treat the envenomation by *Centruroides* [55], and its use is recommended in the case of *Tityus* envenomation [23,48,56]. Despite the usefulness of the antivenom, it is clear that the admission of children in the intensive care unit in addition to the use of antivenom drastically reduced the mortality when compared to the use of antivenom alone or with pharmaceutical support alone in *Tityus* envenoming [23,48,56].

The efficacy of the antivenom in *T. trivittatus* envenomation has been observed experimentally [7,14,57], and clinically [8,15,23,37,41]. Among our data, deaths due to *T. trivittatus* stings are all small children in which the antivenom treatment was not always properly applied. In this regard, it must be pointed out that in the last year a fatality in a teenager occurred in the north-west of the country, the first “non-child” death historically recorded.

The specific antivenom anti-*Tityus* *trivittatus* is produced by immunization of horses using telson homogenates or venom of this scorpion, by the National Institute for Production of Biologics, of the Ministry of Health of Argentina. It is freely distributed in all the country through the Ministry of Health. Nevertheless, other anti-scorpion antivenoms can be used, like Brazilians Antiarachnidic or Soro antiescorpiónico antivenoms (Table 1) produced by immunizing horses with milked venom from *T. serrulatus*. Antiarachnidic antivenom was the only antivenom used before the specific national specific Anti-*Tityus trivittatus* antivenom production. These anti *T. serrulatus* antivenoms (Table 1) can be used since the venoms of *T. serrulatus* ad *T. trivittatus* share some common toxins [58,59] and the principal toxin of *T. trivittatus* venom, a toxin that acts on Na^+^ channels, called gamma toxin, is similar to the correspondent in *T. serrulatus* venom [60].

**Table 1:** Characteristics of the American anti scorpion antivenoms. This table indicates all the antiscorpion antivenoms produced and available in America. These are Anti-*Tityus* in South America (Argentina, Brazil with three producers and Venezuela) and Anti-*Centruroides* in North America (Mexico). The type of presentation, the declared neutralizing potencies, the pharmaceutical characteristics and the immunogens used for their production as well as the method for potency determination, is indicated. Country expresses the country where the antivenom is mostly used independently of where it is produced. All these antivenoms are F(ab’)_2_ fractions or equine immunoglobulins.

Antivenoms									
	Antiescorpión	Soro Antiescorpiónico	Soro Antiaracnídico	Soro Antiescorpiónico	Soro Antiescorpiónico	Suero Antiescorpiónico	Alacramyn	Suero antiescorpión	Anascorp
Country	Argentina	Brazil	Brazil	Brazil	Brazil	Venezuela	Mexico	Mexico	U.S.A.
Producer	INPB-ANLIS “Dr. Carlos G. Malbrán”, Buenos Aires	Instituto Butantan, Sao Paulo	Instituto Butantan, Sao Paulo	Instituto Vital Brazil, Rio de Janeiro	Fundaçao Exequiel Diaz, Belho Horizonte	Universidad Central de Venezuela, Caracas	Insituto Bioclon, S.A. de C.V., Mexico DF	Birmex, S.A. de C.V, Mexico DF	Insituto Bioclon, S.A. de C.V., Mexico DF
Venom used as immunogen	*Tityus trivittatus*	*Tityus serrulatus*	*Tityus serrulatus, Phoneutria sp., Loxosceles gaucho*	*Tityus serrulatus*	*Tityus serrulatus*	*Tityus discrepans*	Pool of *Centruroides* spp.	Pool of *Centruroides* spp.	Pool of *Centruroides* spp.
Animal and route used to determine the potency	mice, i.p.	mice, i.p.	Guinea pigs, s.c.	mice, i.p.	mice, i.p.	mice, i.v.	mice, i.v.	mice, i.v.	mice, i.v.
Neutralizing potency expressed as	LD_50_ of venom neutralized	mg of venom neutralized	MMD of venom neutralized	mg of venom neutralized	mg of venom neutralized	mg of venom neutralized	LD_50 _of venom neutralized	LD_50 _of venom neutralized	LD_50 _of venom neutralized
Potency per vial	over 50 LD_50_	over 5 mg	over 10 MMD	over 5 mg	over 5 mg	1 mg of venom	over 150 LD_50_	over 150 LD_50_	over 150 LD_50_
Presentation	2 mL vial	Ampoule of 5 mL	Ampoule of 5 mL	Ampoule of 5 mL	Ampoule of 5 mL	Ampoule of 5 mL	Lyophilized to be reconstituted (5 mL)	Lyophilized to be reconstituted (5 mL)	Lyophilized to be reconstituted (5 mL)

However, in South America the therapeutics with scorpion antivenoms in absence of local production is not simple, since there are only four countries with a total of seven producers in America, and the immunogens (venoms) for their production, methodology to determine the neutralizing potency and pharmaceutical presentations are very different (see Table 1). Despite these differences, all the anti-*Tityus* antivenom could be therapeutically used in Southern South America because of their cross reactivity among *Tityus* venoms [7,14,61,62]; nevertheless, the absence of clinical assays and the different methods used to determine the neutralizing potencies makes difficult to estimate a correct dose of antivenom when the accident occurs with an heterologous species of *Tityus*. This is important since only in three South American countries this type of antivenom is available, while *Tityus* scorpions are distributed through all South American countries. In addition must be considered that Anti-*Centruroides* antivenom does not neutralize efficiently the venom of *Tityus trivittatus*.

## 7. Comments

Scorpionism in Argentina is a sanitary problem, which in recent year represents around 7000 stings yearly, nearly the 70% of accidents caused by scorpions, snakes and spiders. Furthermore, it causes 2–3 deaths by year [1], being one of the most significant causes of death through envenomation by venomous animals in Argentina [3]. The global sanitary impact is very low when other human pathologies are considered since the number of cases, deaths and lethality are low [2,3,8]. However, the accidents by scorpions are a medical urgency that requires a fast and adequate treatment since the envenomation can cause death if the adequate antivenom and support treatment is not applied fast after the sting. In addition, deaths have increased in the last years. In the period 1994–1998, four deaths were recorded, whereas in the periods 1999–2003 and 2004–2008, eight and 18 deaths were recorded, respectively. This is a disturbing situation due the synanthropic characteristics of this scorpion and the available data on its distribution and increasing findings.

In big cities like Buenos Aires, the highest finding of *T. trivittatus* was in the regions of higher density of inhabitants, and in relation to the old edifications and metro railways of the city, although the findings of this scorpion seem to have expanded in the last years regarding the historical records and our surveillance data [16,17]. Publications on some species of *Tityus* describe that accidents occur in regions where high numbers of scorpions can be found in a city [31,63,64,65] through its adaptation to urban environment [18,19,20,26,66,67,68,] or in determinate regions of a country [19,20,26,68,69,70,71,72,73], and accidents in some Brazilian cities could be associated with old constructions [74,75] and in addition postulate the expansion from nuclei in the cities [76]. Although up to date, we have no data to demonstrate the relationship between density of scorpions and accidents, most of these characteristics are shared by *Tityus trivittatus* in Buenos Aires city. Because of these reasons, the information to the population on measures to prevent the stings and how to proceed in the case of a *T. trivittatus* sting are essential tools in order to diminish the number of cases of envenomation and potential deaths. On the other hand, the instruction to health professionals regarding the correct normative for scorpion stings (*i.e.*, fast application of antivenoms and combined use of antivenom and the intensive care unit), will diminish the deaths, especially in children. In addition, prevention and correct measures of environmental control must be tough on the population in order to lower risk.

Although the sanitary impact of the scorpionism in Argentina has so far been from the impact of some other pathologies, the characteristics of this scorpion and data regarding deaths, and presence of scorpions near human habitats is worrying and needs to be considered in sanitary systems.

Most provinces in Argentina are endemic places for scorpions, and consequently are involved in a potentially very hazardous public health problem.

At present, we are studying the distribution of scorpions, accidents and deaths in 10 big cities of Argentina, where these scorpions have optimum life environmental conditions and food, in order to better understand the occurrence of accidents and envenomations regarding scorpion presence, and to take measures on these subjects of sanitary importance.

## References

[1] De Roodt A.R., Oliveira V., García S.I. (2010). Accidentes por animales venenosos comunicados al Ministerio de Salud de la Nación en el período 2005–2009. Acta Toxicol. Argent..

[2] Casas N., Geffner L., Echenique H., Costa de Oliveira V., de Roodt A.R. (2012). Epidemiologic situation of envenomation by venomous animals in Argentina 2007–2011 period. Toxicon.

[3] De Roodt A.R., Lanari L.C., García S.I., Costa de Oliveira V., Casas N., de Titto E.H. (2013). Accidentes y óbitos por envenenamiento por animales venenosos-ponzoñosos en Argentina en el período 2000–2011. Acta. Toxicol. Argent..

[4] Affilastro A.A. (2005). Estudio monográfico de los escorpiones de la República Argentina. Rev. Ibérica Aracnol..

[5] Acosta L.E., Salomon O.D. (2005). Scorpiones-Escorpiones o alacranes. Artrópodos de Interés Médico en la Argentina.

[6] Acosta L.E., Maury E.A., Morrone J.J., Coscarón S. (1998). Scorpiones. Biodiversidad de Artrópodos argentinos. Una perspectiva biotaxonómica.

[7] de Roodt A.R. (2009). Estudio del Veneno de Algunos Escorpiones de Importancia Médica de la Argentina. Master’s Thesis.

[8] de Roodt A.R., García S.I., Salomón O.D., Segre L., Dolab J.A., Funes R.F., de Titto E.H. (2003). Epidemiological and clinical aspects of scorpionism by *Tityus trivittatus* in Argentina. Toxicon.

[9] Piola J.C., Prada D.B., Waksman J.C., Evangelista M. (2006). Increase mortality and morbidity from *Tityus trivitattus* envenomation in Argentina. Clin. Toxicol..

[10] Maury E.A. (1979). Apuntes para una zoogeografía de la escorpiofauna argentina. Acta Zool. Lilloana.

[11] Maury E.A. (1997). *Tityus trivittatus* en la Argentina. Nuevos datos sobre distribución, partenogénesis, sinantropía y peligrosidad (Escorpiones, Buthidae). Rev. Museo Argent. Cienc. Nat. B. Rivadavia.

[12] De Roodt A.R., Lago N.R., Salomón O.D., Laskowicz R.D., Neder de Román L.E., López R.A., Montero T.E., del V. Vega V. (2009). A new venomous scorpion responsible for severe envenomation in Argentina: Tityus confluens. Toxicon.

[13] Camargo F.J., Ricciardi A. (2000). Sobre la presencia de un escorpión *Tityus serrulatus* Lutz e Mello (Scorpiones, Buthidae) en la ciudad de Corrientes. Comunicaciones Científicas y Tecnológicas 2000, UNNE, Actas.

[14] De Roodt A.R., Coronas F.I.V., Lago N., Gonzalez M.E., Laskowicz R.D., Beltramino J.C., Saavedra S., López R.A., Reati G., Vucharchuc M.G. (2010). General, biochemical and immunological characterization of the venom from the scorpion *Tityus trivittatus* of Argentina. Toxicon.

[15] Docampo P.C., Fernández M.E. (2011). Escorpionismo: Presentación de un posible caso grave ocurrido en la Ciudad de Buenos Aires. Acta Toxicol. Argent..

[16] Laskowicz R., Scarlato E., Lanari L., Blanco G., Lago N., de Roodt A. (2011). Localización geográfica de ejemplares de *Tityus trivittatus* hallados en la ciudad de Buenos Aires. Acta. Toxicol. Argent..

[17] Blanco G., Laskowicz R.D., Scarlatto E., Casas N., Costa de Oliveira V., Lanari L.C., Lago N.R., de Roodt A.R. (2012). Increased incidence of *Tityus trivittatus*. Envenoming in the City of Buenos Aires. Toxicon.

[18] Toscano-Gadea C.A. (2004). Confirmation of parthenogenesis in *Tityus trivittatus* Kraepelin 1898 (Scorpiones, Buthidae). J. Arachnol..

[19] Lourenço W.R. (2008). Parthenogenesis in scorpions: Some histoty—New data. J. Venom. Anim. Toxins Incl. Trop. Dis..

[20] Lourenço W.R., Cloudsley-Thompson J.I., Cuellar O., von Eickstedt V.R.D., Barraviera B., Knox M.B. (1996). The evolution of scorpionism in Brazil in recent years. J. Venom. Anim. Toxins.

[21] Gómez J.P., Otero R. (2007). Eco-epidemiology of scorpions of medical importance in Colombia. Rev. Fac. Nac. Salud Pública.

[22] Salomon O.D., de Roodt A.R. (2001). Escorpiones: Denuncia espontánea en dos centros de referencia en la Ciudad de Buenos Aires, 1997–2000. Medicina Buenos Aires.

[23] Ministerio de Salud de la Nación (2011). Guía de Prevención, Diagnóstico, Tratamiento y Vigilancia Epidemiológica del Envenenamiento por Escorpiones.

[24] Novaes Ramires E., Navarro-Silva M.A., de Assis Marques F., Stoycheva M. (2011). Chemical Control of Spiders and Scorpions in Urban Areas. Pesticides in the Modern World—Pests Control and Pesticides Exposure and Toxicity Assessment.

[25] Nunes C.S., Bevilacqua P.D., Jardim C.C.G. (2000). Demographic and spatial aspects of scorpionic accidents in the Northwest region of Belo Horizonte City, MinasGerais, 1993–1996. Cad. Saúde Pública Rio J..

[26] Ribeiro de Albuquerque C.M., Oliveira Barbosa M., Iannuzzi L. (2009). *Tityus stigmurus* (Thorell, 1876) (Scorpiones; Buthidae): Response to chemical control and understanding of scorpionism among the population. Rev. Soc. Bras. Med. Trop..

[27] Gurtler R.E., Scheigmann N.J., Cecere M.C., Chuit R., Wisinevsky-Colli C. (1993). Comparison of two sampling methods for domestic populations of *Triatoma infestans* in north-west Artentina. Med. Vet. Entomol..

[28] Chandre F., Darriet F., Duchon S., Finot L., Mangiun S., Carnevale P., Guillet P. (2000). Modification of pyrethroid effects associated with kdr mutation in *Anopheles gambiae*. Med. Vet. Entomol..

[29] Ramsey J.M., Salgado L., Cruz-Celis A., López R., Alvear A.L., Espinosa L. (2002). Domestic scorpion control with pyrethroid insecticides in Mexico. Med. Vet. Entomol..

[30] Spirandeli Cruz E.F., Winther Yassuda C.R., Jim J., Barraviera B. (1995). Programa de controle de surto de escorpião *Tityus serrulatus*, Lutz e Mello 1922, no município de Aparecida (Scorpiones, Buthidae). Rev. Soc. Bras. Med. Trop..

[31] Ministério da Saúde (2009). Manual de Controle de Escorpiões.

[32] Holderied M., Korine C., Moritz T. (2011). Hemprich's long-eared bat (Otonycteris hemprichii) as a predator of scorpions: whispering echolocation, passive gleaning and prey selection. J Comp Physiol A Neuroethol Sens Neural Behav Physiol.

[33] Rowe A.H., Rowe M.P. (2006). Risk assessment by grasshopper mice (*Onychomys* spp.) feeding on neurotoxic prey (*Centruroides* spp.). Anim. Behav..

[34] Dor A., Calme S., Henaut Y. (2011). Predatory interactions between *Centruroides scorpions* and the tarantula *Brachypelma vagans*. J. Arachnol..

[35] Martino O., Mathet H., Masini R.D., Ibarra Grasso A., Thompson R., Gondell C., Bosch J., Ministerio de Bienenstar Social (1979). Aracnidismo por escorpiones. Emponzoñamiento Humano Provocado Por Venenos de Origen Animal. Estudio Epidemiológico, Clínico y Experimental.

[36] Esteso S.C., Urtubey N. (1983). Normas básicas de procedimientos, terapéutica y prevención en ofidismo, araneismo y escorpionismo humanos.

[37] Tomassone R. (1994). Emponzoñaamiento por picadura de escorpión. Presentación de cinco casos y revisión bibliográfica. Rev. Col. Médico Pcia. Santa. Fe.

[38] Del Valle Luna M.G., Luna M. (1997). Escorpionismo por *Tityus trivittatus*. Arch. Argent. Pediatr..

[39] Gordillo M.E., Bugliolo A.G., Delloni A. (2000). Escorpionismo en Pediatría. Arch. Argent. Pediatr..

[40] Tomassone R., Vainstub V., Peirano S. (2003). Envenenamiento grave por escorpión en Pediatría. Arch. Argent. Pediatr..

[41] Álvarez Parma J., Palladino C.M. (2010). Scorpion envenomation in Argentina. Arch. Argent. Pediatr..

[42] Abrough F., Elastrous S., Nouira S., Haguiga H., Touzi N., Bouchoucha S. (1999). Serotherapy in scorpion envenomation: A randomized controlled trial. Lancet.

[43] Gueron M., Ovsyshcher I. (1987). What is the treatment for the cardiovascular manifestations of scorpion envenomation?. Toxicon.

[44] Gueron M., Sofer S. (1994). The role of intensivist in the treatment of the cardiovascular manifestations of scorpion envenomation. Toxicon.

[45] Sofer S., Shahak E., Gueron M. (1994). Scorpion envenomation and antivenom therapy. J. Pediatr..

[46] Freire-Maia L. (1994). Pharmacology of *Tityus serrulatus* scorpion venom. Mem. Inst. Butantan.

[47] Freire-Maia L., Campos J.A. (1987). On the treatment of the cardiovascular manifestations of scorpion envenomation. Toxicon.

[48] Freire-Maia L., Campos J.A., Amaral C.F.S. (1994). Approaches to the treatment of scorpion envenoming. Toxicon.

[49] Dehesa-Dávila M., Possani L.D. (1994). Scorpionism and Serotherapy in Mexico. Toxicon.

[50] Possani L.D. (2000). Antivenom for scorpion sting. Lancet.

[51] Ghalim N., El-Hafny B., Sebti F., Heikel J., Lazar N., Moustanir R., Benslimane A. (2000). Scorpion envenomation and serotherapy in Morocco. Am. J. Trop. Med. Hyg..

[52] Ismail M. (1994). The treatment of the scorpion envenoming syndrome: The Saudi experience with serotherapy. Toxicon.

[53] Cupo P., de Azevedo-Marques M.M, Hering S.E. (2003). Acidentes por Animais Peçonhentos: Escorpioes e Aranhas. Medicina Riberao Preto.

[54] Osnaya-Romero N., de Jesus Medina-Hernández T., Flores-Hernández S.S., Leon-Rojas G. (2001). Clinical symptoms observed in children envenomed by scorpion stings, at the children’s hospital from the state of Morelos, Mexico. Toxicon.

[55] Boyer L.V., Theodorou A.A., Berg R.A., Joanne Mallie R.N. (2009). For Arizona Envenomation Investigators; Chávez-Mendez, A.; García-Ubbelohde, W.; Hardiman, S.; Alagón A. Antivenom for critically ill children with neurotoxicity from scorpion stings. N. Engl. J. Med..

[56] Cupo P., de Azevedo-Marques M.M., Hering S.E., Cardoso J.L.C., Fan H.W., França F.O.S., Wen F.H., Málaque C.M.S., Haddad V. (2003). Escorpionismo. Animais Peçonhenetos no Brasil–Biologia, Clínica e terapêutica dos Acidentes.

[57] De Roodt A.R., Gimeno E., Portiansky E., Varni L., Dolab J.A., Segre L., Litwin S., Vidal J.C. (2001). A study on the experimental envenomation in mice with the venom of *Tityus trivittatus* Kraepelin 1898 (Scorpions, Buthidae) captured in Argentina. J. Nat. Toxins.

[58] Coronas F.E., de Roodt A.R., Olamendi Portugal T., Zamudio F.Z., Batista C.B.F., Gómez Lagunas F., Posani L.D. (2003). Disulfide bridges and blockage of Shaker B K^+^—Channels by another butantoxin peptide purified from the Argentinean scorpion *Tityus trivittatus*. Toxicon.

[59] Abdel-Mottaleb Y., Coronas F.V., de Roodt A.R., Possani L.D., Tytgat J. (2006). A novel toxin from the venom of the scorpion *Tityus trivittatus*, is the first member of a new α-KTX subfamily. FEBS Lett..

[60] Coronas F.I., Diego-García E., Zamudio F., de Roodt A.R., Restano-Cassulini R., Possani L.D. (2007). Cloning and Peptide Sequencing of a Gamma-Like Toxin from the Argentinian Scorpion Tityus trivittatus.

[61] Nishikawa A.K., Caricati C.P., Lima M.L., dos Santos M.C., Kipnis T.L., Eickstedt V.R., Knysak I., da Silva M.H., Higashi H.G., da silva W.D. (1994). Antigenic cross-reactivity among the venoms from several species of Brazilian scorpions. Toxicon.

[62] Fundaçao Nacional de Saúde, Escorpionismo (1999). Manual de Diagnóstico e Tratamento de Acidentes por Animais Peçonhentos.

[63] Morais Lima A.L., Alves de Lima J., da Silva Souto M.C., da Costa Lopes T.F., da Silva Torres U.P., Campos Cavalcanti M.A. (2011). Spatial distribution and epidemiological profile of scorpion accidents in Natal/RN. ConSci. Saúde.

[64] Monteiro de Amorim A., Martins Carvalho F., Lira-da-Silva R., Kobler Brazil T. (2003). Scorpion sting in an area of Nordeste de Amaralina, Salvador, Bahia, Brazil. Rev. Soc. Bras. Med. Trop..

[65] Ribeiro de Albuquerque C.M., de Lima Santana Neto P., Porto Amorim M.L., Campos Vidal Pires S. (2013). Pediatric epidemiological aspects of scorpionism and report on fatal cases from *Tityus stigmurus stings* (Scorpiones: Buthidae) in State of Pernambuco, Brazil. Rev. Soc. Bras. Med. Trop..

[66] Larcher Carneiro Santos P., Martins F.J., de Cássia Padula Alves Vieira R., Ribeiro L.C., Beloti Barreto B., Rezende Barbosa N. (2010). Características dos accidentes escorpiónicos em Juiz de Fora–MG. Rev. APS.

[67] Lira A.F., Souza A.M., Silva Filho A.A., Albuquerque C.M. (2013). Spatio-temporal microhabitat use by two co-ocurring species of scorpions in Atlantic rainforest in Brazil. Zoology (Jena).

[68] Lourenço W.R., da Silva E.A. (2007). New evidence for a disrupted distribution pattern of the *Tityus confluens* complex, with the description of a new wpecies from the State of Pará, Brazil (Scorpiones, Buthidae). Amazoniana.

[69] Von Eickstedt V.R.D., Ribeiro L.A., Candido D.M., Albuquerque M.J., Jorge M.T. (1996). Evolution of scorpionism by *Tityus bahiensis* (Perty) and *Tityus serrulatus* (Lutz and Mello) and geographical distribution of the two species in the state of Sao Paulo, Brazil. J. Venom. Anim. Toxins.

[70] Mazzei de Davila C.A., Parra M., Feunmayor A., Salgar N., González Z., Davila D.F. (1997). Scorpion envenomation in Merida, Venezuela. Toxicon.

[71] Gómez J.P., Quintana J.C., Arbeláez P., Fernández J., Silva J.F., Barona J., Gutiérrez J.C., Díaz A., Otero R. (2010). Picaduras por escorpión *Tityus asthenes* en Mutatá, Colombia: Aspectos epidemiológicos, clínicos y toxinológicos. Biomédica.

[72] Borges A., de Sousa L. (2006). Escorpionismo en Venezuela: Una aproximación molecular, inmunológica y epidemiológica para su estudio. Rev. Fac. Farm..

[73] De Sousa L., Parrilla-Alvarez P., Quiroga M. (2000). An epidemiological review of scorpion stings in Venezuela: The northeastern region. J. Venom. Anim. Toxins.

[74] Martins Soares M.R., Schetini de Azevedo C., de Maria M. (2002). Scorpionism in Belo Horizonte, MG: A retrospective study. Rev. Soc. Bras. Med. Trop..

[75] Guerra C.M.N., Carvalho L.F.A., Colosino E.A., Freire H.B.M. (2008). Analysis of variables related to fatal outcomes of scorpion envenomation in children and adolescents in the state of Minas Gerais, Brazil, from 2001 to 2005. J. Pediatr..

[76] De Andrade H.P., Pasquletto A. (2002). Epidemia urbana de *Tityus serrulatus* no município de Trindade (GO). Rev. Univ. Católica Goiás.

